# Effect of symbiotic fungi-*Armillaria gallica* on the yield of *Gastrodia elata* Bl. and insight into the response of soil microbial community

**DOI:** 10.3389/fmicb.2023.1233555

**Published:** 2023-09-07

**Authors:** Yanhong Wang, Jiao Xu, Qingsong Yuan, Lanping Guo, Chenghong Xiao, Changgui Yang, Liangyuan Li, Weike Jiang, Tao Zhou

**Affiliations:** ^1^Resource Institute for Chinese & Ethnic Materia Medica, Guizhou University of Traditional Chinese Medicine, Guiyang, China; ^2^National Resource Center for Chinese Materia Medica, China Academy of Chinese Medical Sciences, Beijing, China

**Keywords:** high-throughput sequencing, symbiotic fungi, microbial community, *Armillaria*, *Gastrodia elata* Bl.

## Abstract

*Armillaria* members play important roles in the nutrient supply and growth modulation of *Gastrodia elata* Bl., and they will undergo severe competition with native soil organisms before colonization and become symbiotic with *G. elata*. Unraveling the response of soil microbial organisms to symbiotic fungi will open up new avenues to illustrate the biological mechanisms driving *G. elata*’s benefit from *Armillaria*. For this purpose, *Armillaria* strains from four main *G. elata* production areas in China were collected, identified, and co-planted with *G. elata* in Guizhou Province. The result of the phylogenetic tree indicated that the four *Armillaria* strains shared the shortest clade with *Armillaria gallica.* The yields of *G. elata* were compared to uncover the potential role of these *A. gallica* strains. Soil microbial DNA was extracted and sequenced using Illumina sequencing of 16S and ITS rRNA gene amplicons to decipher the changes of soil bacterial and fungal communities arising from *A. gallica* strains. The yield of *G. elata* symbiosis with the YN strain (*A. gallica* collected from Yunnan) was four times higher than that of the GZ strain (*A. gallica* collected from Guizhou) and nearly two times higher than that of the AH and SX strains (*A. gallica* collected from Shanxi and Anhui). We found that the GZ strain induced changes in the bacterial community, while the YN strain mainly caused changes in the fungal community. Similar patterns were identified in non-metric multidimensional scaling analysis, in which the GZ strain greatly separated from others in bacterial structure, while the YN strain caused significant separation from other strains in fungal structure. This current study revealed the assembly and response of the soil microbial community to *A. gallica* strains and suggested that exotic strains of *A. gallica* might be helpful in improving the yield of *G. elata* by inducing changes in the soil fungal community.

## Introduction

Mycorrhizal symbiosis is found in over 80% of extant land plant species (Wang et al., 2017; Feijen et al., 2018). Orchidaceae showing mycorrhizal association with fungi are essential for seed germination and plant growth (Currah et al., 1997; Brundrett, 2002). It is believed that the ability to recruit saprotrophic lineages of wood and litter-inhibiting fungi is considered instrumental for plant adaptation to various ecological challenges, and many symbiotic fungi have coevolved with plants and showed a high degree of host specificity (Raaijmakers et al., 2009; Crowther et al., 2012). A well-known and highly specific alliance example is *Gastrodia elata* Bl. and *Armillaria* (Yuan et al., 2018; Camprubi et al., 2020; Xu et al., 2021). *G. elata*, also called Tianma in Chinese, is a perennial herb in the Orchidaceae family and one of the most valuable traditional Chinese medicines. Being one of the heterotrophic plants without chlorophyll, the growth and development of *G. elata* require symbiosis with *Armillaria* for nutrient supply since the protocorm stage (Yuan et al., 2018). The *Armillaria* members impose a significant impact on the yield and quality of *G. elata*; therefore, the isolation and identification of excellent *Armillaria* strains are of great significance (Yu et al., 2022).

Members of *Armillaria* generally form linear mycelial organs involved in wood decay and nutrient absorption. Evidence indicates that rhizomorphs of wood-decay species can absorb inorganic nutrients from the external environment (Clipson et al., 1987; Cairney et al., 1988), and the melanized layer of rhizomorphs generally contains calcium, which can provide chemical defense and natural controls (Porter et al., 2022). However, recent studies observed increased diversity of bacteria and fungi during the juvenile tuber to mature tuber periods of *G. elata*, which implied that *Armillaria mellea* can affect the structure of the microbial community associated with *G. elata* and greatly reduce the antifungal and antibacterial activities as a symbiotic association with its host is established (Yuan et al., 2018).

Soil contains large amounts of microbial biomass, and its microbial communities are highly diverse (Torsvik and Vres, 2002; Fierer, 2017). Diversity, structure, and composition of the soil microbial community are central issues in agricultural management, as this information is crucial for understanding and predicting the role played by organisms in maintaining plant health and production (Berendsen et al., 2012). Biotic factors, such as plant cultivars, strongly influence the composition of the soil rhizosphere microbiota and control the assembly of root-associated microbiotas (Tkacz et al., 2015; Zhou and Fong, 2021). Normally, plant cultivars impose an effect on the rhizosphere microbiota by investing a huge amount of root exudates and providing sources of carbonaceous compounds (Bulgarelli et al., 2013). Symbiotic fungi also have the ability to influence soil microbes; however, unlike plants, secretions of fungi through mycelia or rhizomorphs might inhibit the growth of others (Collins et al., 2013). Studies of changes in microbial community composition in response to symbiotic fungi can foster a better understanding of *Armillaria* utilization in the future.

The geographical areas of *G. elata* cultivation are mainly spread over the Ta-pieh Mountains, Qinling-Daba Mountains, and Yunnan-Kweichow Plateau in China. These sites are typically among the regions with sufficient rainfall and variable air temperatures in the summer. As global climate change continues and frequent commercial trade occurs among planting regions, it is incredibly significant to choose alternative *Armillaria* strains that can adapt to the diverse cultivation conditions in the process of *G. elata* artificial cultivation. However, a knowledge gap exists regarding to what extent *G. elata* will benefit from and how soil microbial communities vary with different *Armillaria* strains. For this purpose, four accessions of *Armillaria* strains were collected around China and tested in Guizhou Province. The genetic relationship of *Armillaria* strains was identified, and the yields of *G. elata* and soil microbial community dynamic were compared. Findings from this current study might be helpful in optimizing our strategies to utilize symbiotic fungi and *Armillaria* strains.

## Materials and methods

### Collection and identification of *Armillaria* strains

In this study, rhizomorphs of *Armillaria* were collected from three wild conditions in Lu’an (115°22′E, 31°06′N) Anhui Province, Xiaocaoba (103°51′E, 27°16′N) Yunnan Province, and Bijie (105°00′E, 27°14′N) Guizhou Province, and one commercial *Armillaria* strain was from Ningqiang (106°30′E, 32°37′N) Shanxi Province (Supplementary Figure S1). All these provinces are the main producing regions of *G. elata*. First, all the rhizomorphs of *Armillaria* were rinsed with running water and soaked with 75% ethanol for 45 s, followed by 1% NaOCl for 6 min. Then, the rhizomorphs were rinsed with sterilized water 3–4 times and dried with microbe-free filter paper. Finally, the cortices of rhizomorphs were removed with a sterilized knife, and the mycelia were lightly pulled out. Equal sizes and sections of mycelia for each *Armillaria* were inoculated in potato dextrose agar (PDA) solid medium and cultured for 20 days. In total, four *Armillaria* isolates, in accordance with collection sites, were obtained in the laboratory and named as follows: AH strain, SX strain, YN strain, and GZ stain.

The DNA of *Armillaria* isolates was extracted using the first generation with the Ezup Column Fungi Genomic DNA Purification Kit (Sangon Biotech, Shanghai, China). Then, the intergenic spacer (IGS) regions of ribosomal RNA genes were amplified with primers CNL12 (5’-CTGAACGCCTCTAAGTCAG-3′) and 5SA (5’-CAGAGTCCTATGGCCGTGGAT-3′). The amplicons were subjected to Sanger sequencing, and gene sequence comparisons were done with the basic local alignment search (BLAST) homology comparison.^1^ The purified *Armillaria* strains were kept on medium for one generation, then the second generation was used as the primary strain and enlarged by cultivating in sterilized packets with oat bran and cornstalk as nutrient sources. The conditions of *Armillaria* strain packets used for *G. elata* artificial cultivation were presented with graphs in Supplementary Figure S2.

### Experiment design and sampling procedure

The site of *G. elata* artificial cultivation was located at Gonglongping National Forest Park in Qixingguan district, Bijie, Guizhou Province, China (105°00′38′′E, 27°14′43′′N), 1781 m above sea level. The mean annual temperature and precipitation in this area are 12.8°C and 998 mm, respectively. The soil is classified as brown loam (FAO-UNESCO, 1974).

The trial was performed at a single field site that was newly explored to ensure a uniform growth condition among repetitions. The cultivation of *G. elata* was carried out according to the WHO guidelines on good agricultural and collection practices (GACP) for medicinal plants.^2^ In the beginning, holes with a size of 80 cm × 50 cm × 30–50 cm (length × width × depth) were dug in the field. Then, 5–8 pieces of chestnut wood (Ф = 5–10 cm, length = 30 cm) that were fully infected with *Armillaria* strains were neatly laid out on the bottom of each hole. Subsequently, 0.1 kg of juvenile tubers of *G. elata* were put evenly on the chestnut wood and crushed with twigs and leaves that were infected with *Armillaria* strains. Finally, the soil was filled back into the holes and covered with straw or leaves (Yuan et al., 2020). The chestnut wood, twigs, and leaves that were infected with *Armillaria* strains were prepared ahead of cultivation, and the strains used for the chestnut wood, twig, and leaf infection were in a one-to-one relationship. There are four strains of *Armillaria* used in this trial in total, namely, AH strain, SX strain, YN strain, and GZ strain; each of them was replicated in three.

The cultivation was processed in March 2021, and the sampling was conducted in November 2021. When sampling, the twigs and leaves were removed gently without disturbing the soil covered by *G. elata*. A tensiometer was used to check the tensile strength of mycelia. The conditions of *Armillaria* mycelia at harvest time can be found in Supplementary Figure S3. At first, *Armillaria* strains tightly connected with chestnut wood and *G. elata* were picked out; subsequently, a section of *Armillaria* rhizomorph was hung on the hook of the tensiometer; then, the other side of the rhizomorph was pulled until it broke; and the real-time data on the tensiometer were recorded. Eight mycelia within one hole were randomly selected for statistical analysis. Soils around mature *G. elata* tubers were collected. Briefly, the soils loosely attached to *G. elata* were carefully collected into a 50-ml centrifuge tube with 20 mL of sterile water. After vortex mixing at 200 rpm for 20 min and centrifuging at 11,000 rpm for 10 min, the deposit was maintained at −80°C for subsequent soil microbial DNA extraction. Finally, all fresh tubes including mature and juvenile tubes of *G. elata* within one hole were collected and weighed, and the data were recorded.

### Soil microbial DNA extraction, amplification, and Illumina sequencing

Soil microbial DNA was extracted using the E.Z.N.A.® soil DNA Kit (Omega Bio-tek, Norcross, GA, United States). The 16S rRNA gene was amplified with primers (F: ACTCCTACGGG AGGCAGCA, R: GGACTACHVGGGTWTCT AAT), and the ITS rRNA gene was amplified with primers (F: CTTGGTCATTTA GAGGAAGTAA, R: GCTGCGTTCTTCATCGATGC) with a 12 nt unique barcode at the 5′ end. The PCR reactions were performed in triplicate in 20-μl mixtures, containing 1 μL of 10-fold diluted DNA, 10 μL of SYBR^®^ Premix Ex Taq (Tli RNase H Plus, 2×, Takara Bio, Japan), 1 μL of forward primer and 1 μL of reverse primer at 10 mM, and 7 μL of Milli-Q water. The PCR products were detected by electrophoresis in a 2% agarose gel, and then purified using the agarose gel DNA purification kit (Takara, Dalian, China). Furthermore, equal amounts of purified amplicons were pooled in equimolar amounts and paired-end sequenced by the Illumina NovaSeq platform at Biomarker Technologies Co., Ltd. (Beijing, China).

### Processing of sequencing data and bioinformatics analysis of microbiome sequencing

Raw reads were quality-filtered using Trimmomatic (v0.33) and then clean reads were obtained with Cutadapt (1.9.1) by identifying and removing the primer sequences. Usearch^3^ was used to merge the paired-end reads into a tag from high-quality clean reads. The operational taxonomic units (OTUs) were defined at the 97% similarity level by Usearch (Edgar, 2013) with the remaining unique sequences. At 70% of the confidence threshold, the taxonomic identity of phylotypes was classified using the Ribosomal Database Project RDP Classifier^4^, the 16S rRNA gene sequence was analyzed with the Silva (SSU123) 16S rRNA database, and the ITS rRNA gene sequence was analyzed against the UNITE database (Version 6).

The Venn diagrams were constructed to visualize shared and unique OTUs among samples. Based on the calculated Bray–Curtis distance, the NMDS analysis was conducted to investigate the variation of bacterial and fungal community structure among treatments. To assess the significance of the difference in the bacterial and fungal community structure among different *Armillaria* strains, permutational multivariate ANOVA (PERMANOVA) was conducted using the ADONIS tests in the R vegan package (ADONIS; Oksanen et al., 2014). Based on the pairwise Spearman method and correlation coefficient with a threshold value of *r* ≥ 0.5, the co-occurrence network analysis of bacteria and fungi was calculated at a *p* < 0.5 significant level, and the analysis and visualization were performed on the platform BMKCloud.^5^

The statistical significance of the yield of *G. elata*, tensile strength of *Armillaria* mycelia, OTU numbers, alpha diversity of bacterial and fungal communities, and relative abundance at the genus level among different *Armillaria* strains were tested using a one-way analysis of variance (ANOVA) and Duncan’s multiple range test at a 0.05 significance level (SPSS 20.0 for Windows, IBM, Chicago, IL, United States). The IGS sequence comparisons of *Armillaria* strains were done with BLAST (see footnote 1), and a phylogenetic tree based on gene sequence comparisons was constructed with Mega (version 11, Tamura et al., 2021).

## Results

### Identification of four Armillaria strains

**Figure 1:**
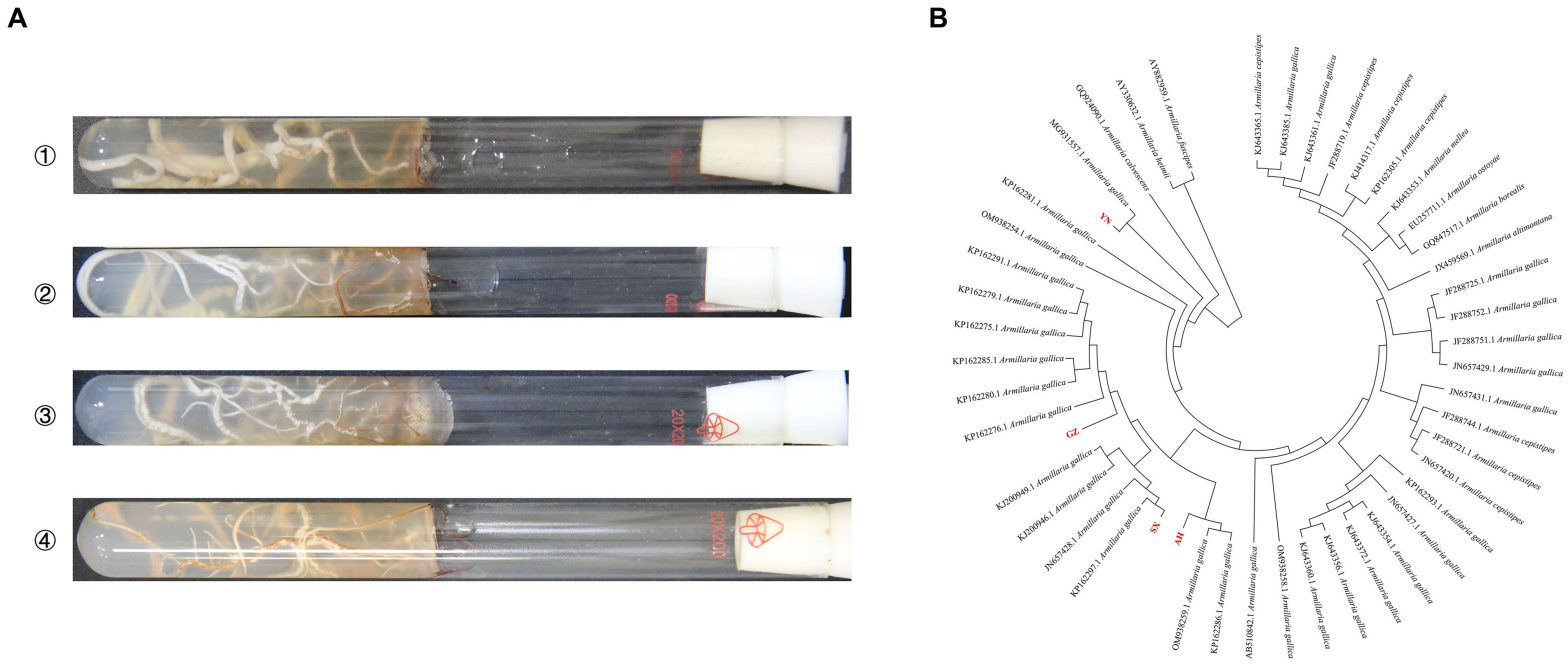
Incubation and identification of *Armillaria* isolates. **(A)** Mycelial morphology of four isolates on PDA medium at 23°C for 20 days (①: YN, ②: AH, ③: SX, ④: GZ). **(B)** Phylogenetic analysis of four *Armillaria* isolates based on the intergenic spacer.

The gene sequence comparisons of intergenic spacers were done with BLAST homology comparison to classify the origins of *Armillaria* strains. The results of BLAST indicated that AH, SX, and GZ strains shared more than 98% homology with *A. gallica*, while the YN strain shared 82% with *A. gallica* in sequences producing significant alignments. The phylogenetic analysis based on sequences showed that all the *Armillaria* strains were clustered in three main clades, and the four strains and *A. gallica* had very close genetic relationships (Figure 1).

### Yields of *Gastrodia elata* symbiosis with *Armillaria gallica* strains and the tensile strength of *Armillaria gallica* mycelia at the harvest time

**Figure 2:**
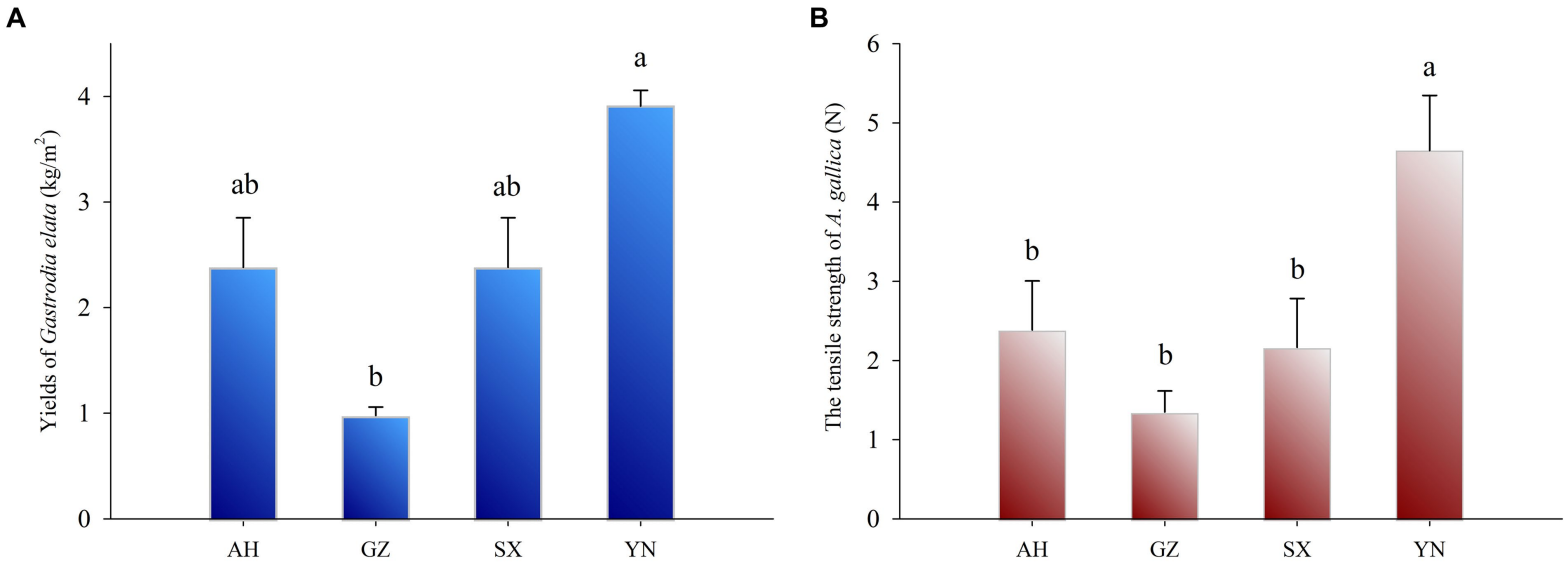
Yields of *G. eleta* symbiosis with *Armillaria* strains from different production areas for 8 months **(A)** and the tensile strength of *Armillaria* strains at harvest time **(B)**. Lowercase letters a, b, and c within one plot indicate significant differences among treatments, determined using the one-way ANOVA and Duncan’s multiple range test (*p* < 0.05).

The yield of *G. elata* symbiosis with the YN strain was the highest (3.91 kg·m^−2^) (Figure 2A) and significantly higher than that of other strains (*p* < 0.05). In addition, the yield of *G. elata* symbiosis with SX and AH was both 2.38 kg·m^−2^ and markedly higher compared with the yield of *G. elata* symbiosis with the GZ strain, which was 0.98 kg·m^−2^ (*p* < 0.05).

To assess the ability of nutrient supply in different *A. gallica* strains, we tested the tensile strength of *A. gallica* mycelia at harvest time using a tensiometer. The tensile strength of the YN strain was significantly higher (*p* < 0.05) than that of the SX, AH, and GZ strains (Figure 2B), and strong growth of mycelia was observed in the YN strain compared with others (Figure 1A). However, there was no significant difference in the tensile strength among the SX, AH, and GZ strains.

### Numbers of OTUs and Venn analysis across samples

The next-generation sequencing generated 959,747 pairs of reads for 16S rRNA genes and 959,978 pairs of reads for ITS rRNA genes across all samples. After rarefication to the minimum average, 79,764 and 79,666 clean reads were obtained for bacteria and fungi, respectively. Based on 97% similarity, 19,265 and 7,588 OTUs were identified for the bacterial and fungal communities, respectively.

**Figure 3:**
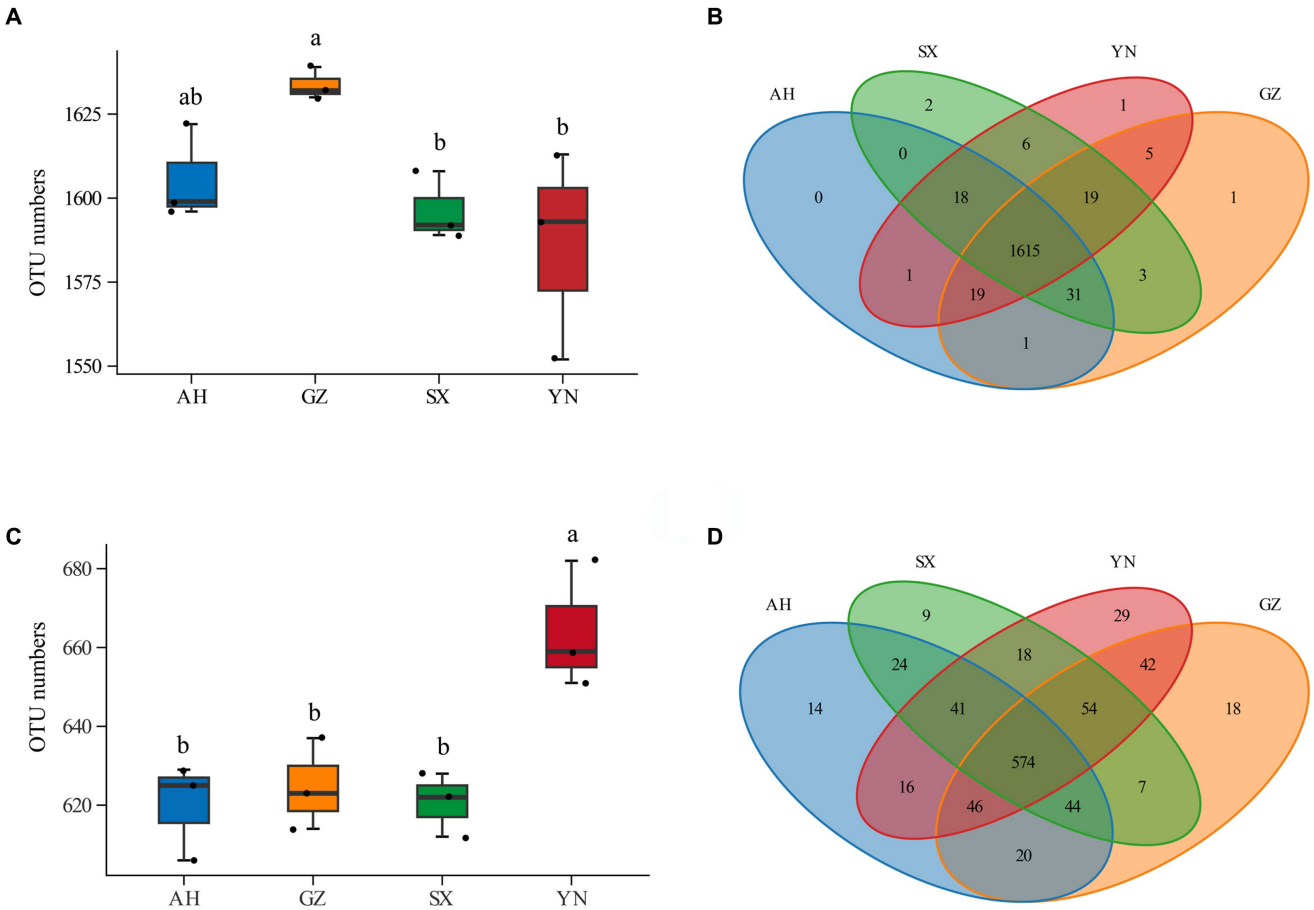
OTU numbers and Venn analysis of bacterial and fungal communities in soils around *G. eleta* symbiosis with *Armillaria* strains. **(A,B)** Represent OTU numbers and the Venn diagram of the bacterial community; **(C,D)** represent OTU numbers and the Venn diagram of the fungal community. Lowercase letters a and b indicate significant differences among different soils, determined using the one-way ANOVA and Duncan’s multiple range test (*p* < 0.05).

Compared with the SX and YN strains, the GZ strain resulted in increases in soil bacterial OTU numbers (Figure 3A). Compared with others, the YN strain led to significantly higher OTU numbers in soil fungal community (Figure 3C). The Venn diagram analysis showed that there were large numbers of OTUs shared across samples, while less peculiar OTUs were spread in the bacterial community (Figure 3B). In the fungal community, the numbers of peculiar OTUs in soil planted with the YN strain were much higher than those of the AH, SX, and GZ strains, and more numbers of shared OTUs were found across treatments (Figure 3D).

### Dynamics of soil microbial community diversity and structure in response to different *Armillaria gallica* strains

**Figure 4:**
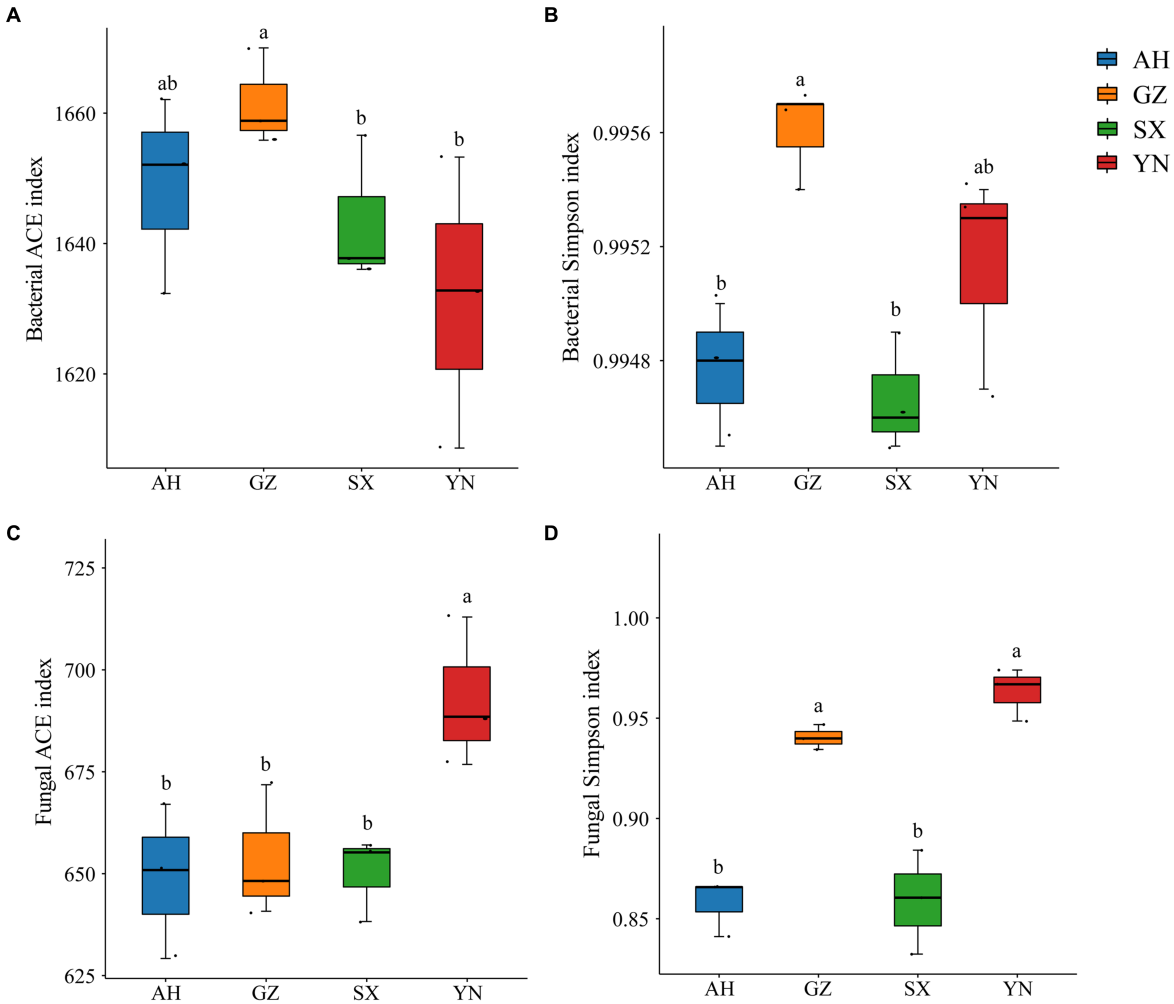
Alfa diversity indices of bacterial and fungal communities in soils around *G. eleta* symbiosis with *Armillaria* strains. **(A,B)** Represent the Ace and Simpson indexes of the bacterial community; **(C,D)** represent the Ace and Simpson indexes of the fungal community. Lowercase letters a and b indicate significant differences among different soils, determined using the one-way ANOVA and Duncan’s multiple range test (*p* < 0.05).

We analyzed the response of the soil bacterial and fungal communities to different *A. gallica* strains through Illumina sequencing. The diversity and richness of bacteria and fungi varied among different *A. gallica* strains and showed different patterns (Figure 4). In particular, the ACE and Simpson indexes of bacterial diversity in soil planted with the GZ strain significantly increased compared with those of other strains (Figures 4A,B), whereas the changes in fungal diversity indexes were different from those of bacteria. Specifically, the ACE and Simpson indexes tended to increase in soil planted with the YN strain, which showed significant differences with others (Figures 4C,D). Overall, the α diversity of the soil bacterial community was significantly influenced by the GZ strain, while that of the soil fungal community was markedly altered by the YN strain.

**Figure 5:**
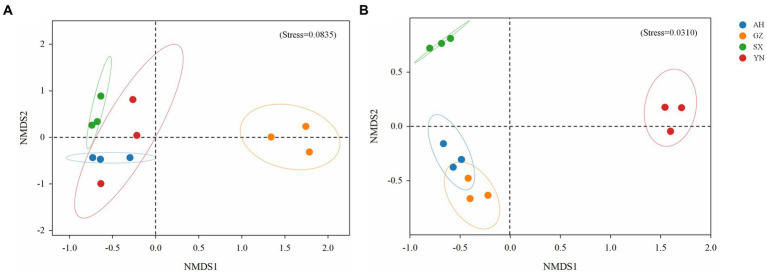
Non-metric multidimensional scaling (NMDS) analysis of bacterial **(A)** and fungal **(B)** communities in soils around *G. eleta* symbiosis with *Armillaria* strains, calculated based on Bray-Curtis distance.

Similarly, the community structure of bacteria and fungi significantly changed (Figure 5). The result of NMDS analysis showed that the bacterial community in soil planted with the GZ strain clustered together and was greatly separated from the other strains (*R*^2^ = 0.309, *p* < 0.01) (Figure 5A). Meanwhile, the fungal community structures of YN and SX strains were greatly separated from the others (*R*^2^ = 0.401, *p* < 0.01), while no significant difference was observed between AH and GZ strains (*p* = 0.1) (Figure 5B).

### Changes of microbial composition in different *Armillaria gallica* strains

**Figure 6:**
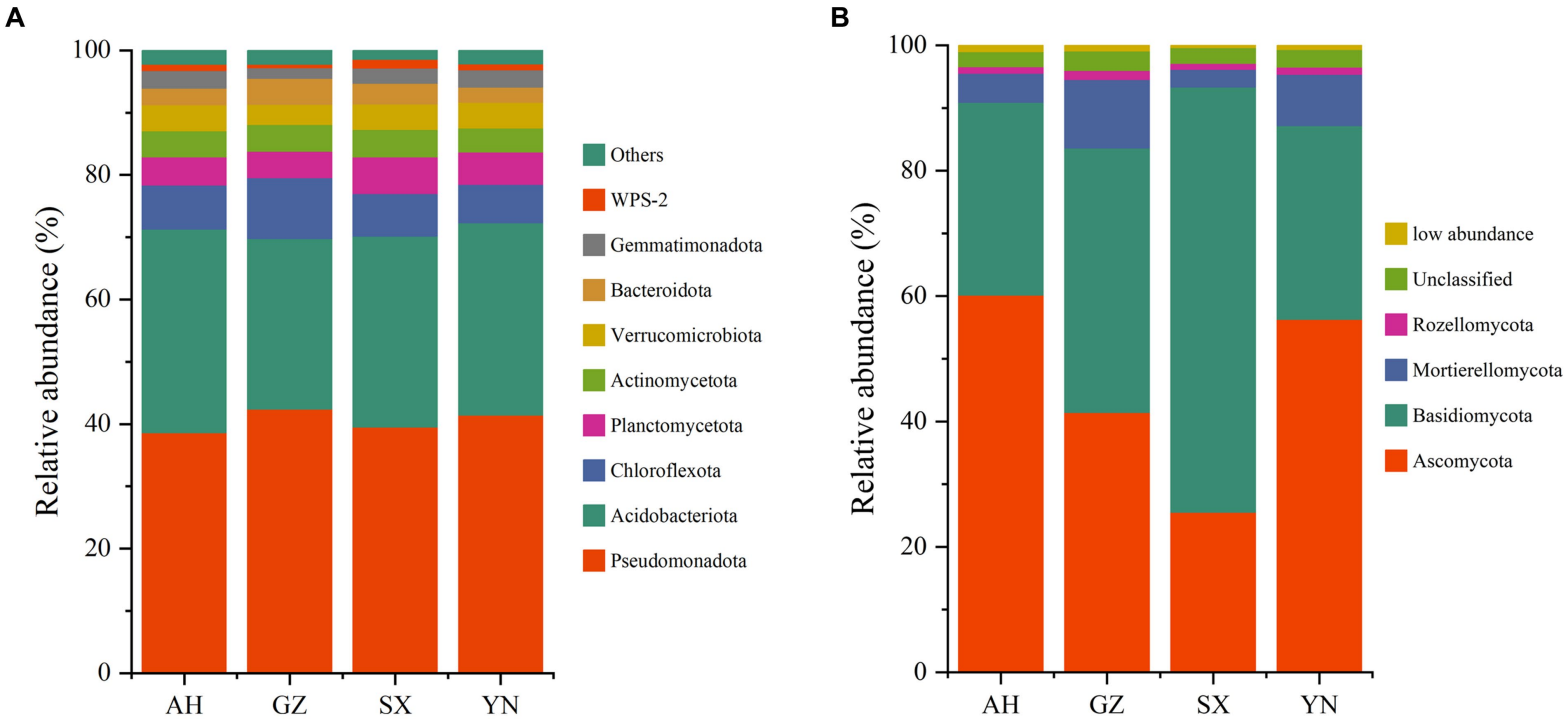
The bacterial **(A)** and fungal **(B)** community compositions in soils around *G. eleta* symbiosis with *Armillaria* strains at the level of phylum classification.

The bacterial community was predominated by 9 phyla with a minor proportion of others (1.5–2.3%). The phyla Pseudomonadota (38.57–42.34%), Acidobacteriota (27.39–32.69%), and Chloroflexota (6.12–9.77%) were dominant members, followed by Planctomycetota (4.26–5.91%), Actinomycetota (3.83–4.38%), Verrucomicrobiota (3.21–4.16%), Bacteroidota (2.43–4.15%), Gemmatimonadota (1.73–2.81%), and WPS-2 (0.53–1.41%) (Figure 6A). None of the *A. gallica* strains induced significant changes in the relative abundances of Pseudomonadota (*p* = 0.344). However, compared with other strains, planting GZ strains significantly increased the abundances of Chloroflexota (*p* < 0.05) while significantly decreasing the abundances of Acidobacteriota (*p* < 0.01).

In the total ITS rRNA amplicons, OTUs in all samples were classified into four phyla, namely, Ascomycota (25.48–60.13%), Basidiomycota (30.73–67.80%), Mortierellomycota (2.85–10.96%), and Rozellomycota (0.95–1.44%), besides the unclassified (2.42–3.09%) and the sum of relative abundances that were lower than 1% (0.40–1.04% in total) (Figure 6B). Notably, the significant role of *A. gallica* strains in affecting the rhizomicrobiome in three dominant fungal phyla, i.e., Ascomycota, Basidiomycota, and Mortierellomycota, was detectable (*p* < 0.05) (Figure 6B). Specifically, AH and YN strains significantly increased the relative abundance of Ascomycota and Mortierellomycota (*p* < 0.01) while decreasing the relative abundance of Basidiomycota compared with other strains.

**Figure 7:**
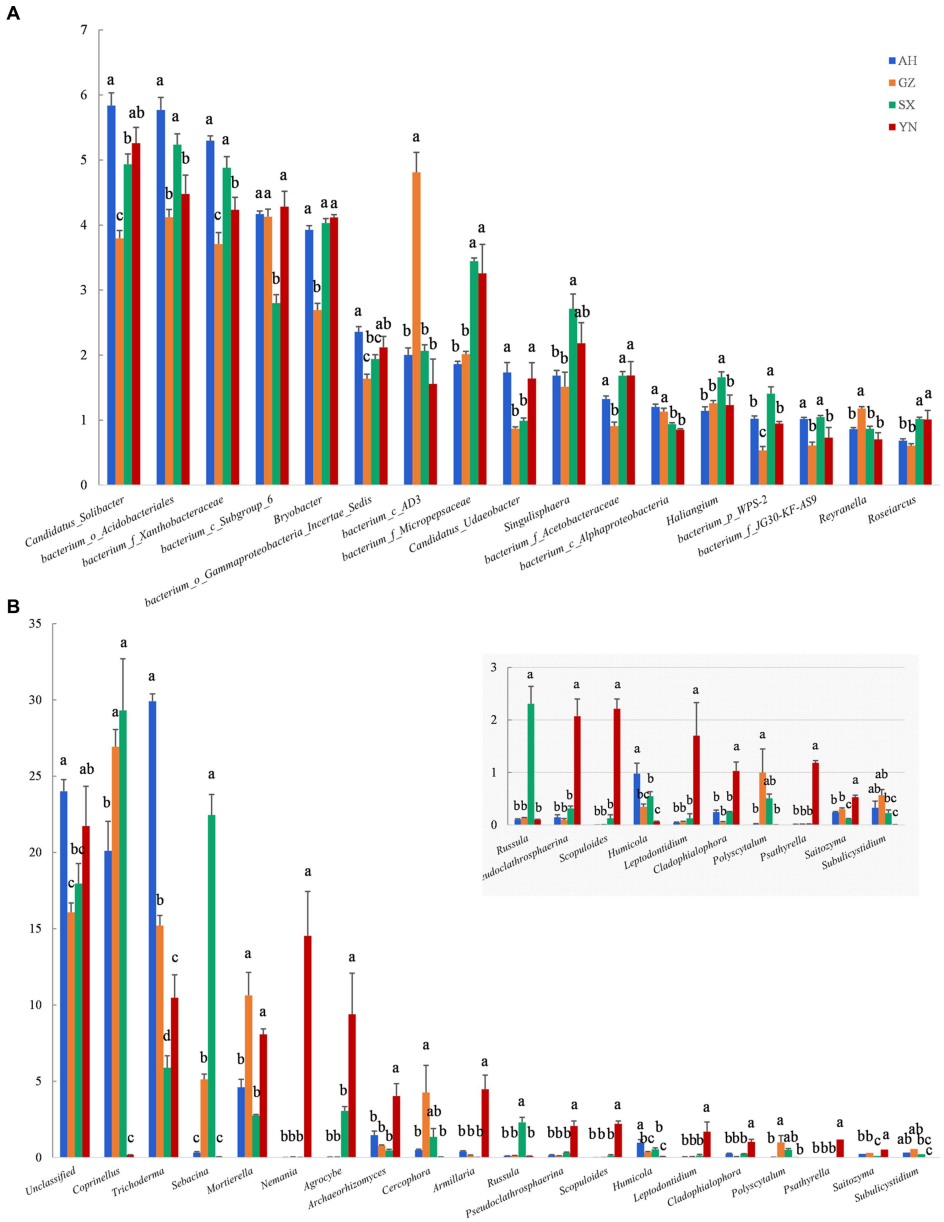
Relative abundance of soil bacterial **(A)** and fungal **(B)** communities in soils around *G. eleta* symbiosis with *Armillaria* strains at the level of genus classification. Lowercase letters a, b, and c within one genus taxa indicate significant differences among treatments, determined using the one-way ANOVA and Duncan’s multiple range test (*p* < 0.05).

At the genus level, the number of genera greatly responded to different *A. gallica* strains. Compared with others, relative abundances of most genera of bacteria were mainly reduced in soil planted with GZ strain, except for the *bacterium_c_AD3*, *bacterium_c_Alphaproteobacteria*, and *Reyranella*, whose relative abundances were significantly increased by GZ strain (*p* < 0.05) (Figure 7A). However, most fungal groups were enriched by the YN strain. For example, the genera with increased relative abundances in response to the YN strain included *Mortierella*, *Nemania*, *Agrocybe*, *Archaeorhizomyces*, *Armillaria*, *Pseudoclathrosphaerina*, *Scopuloides*, *Leptodontidium*, *Cladophialophora*, *Psathyrella*, and *Saitozyma* (*p* < 0.05) (Figure 7B). Other genera showed negative responses to the YN strain, including *Coprinellus*, *Sebacina*, and *Russula*; however, their relative abundances were significantly increased by the SX strain (*p* < 0.05).

### Co-occurrence patterns of bacterial and fungal communities responding to different *Armillaria gallica* strains

**Figure 8:**
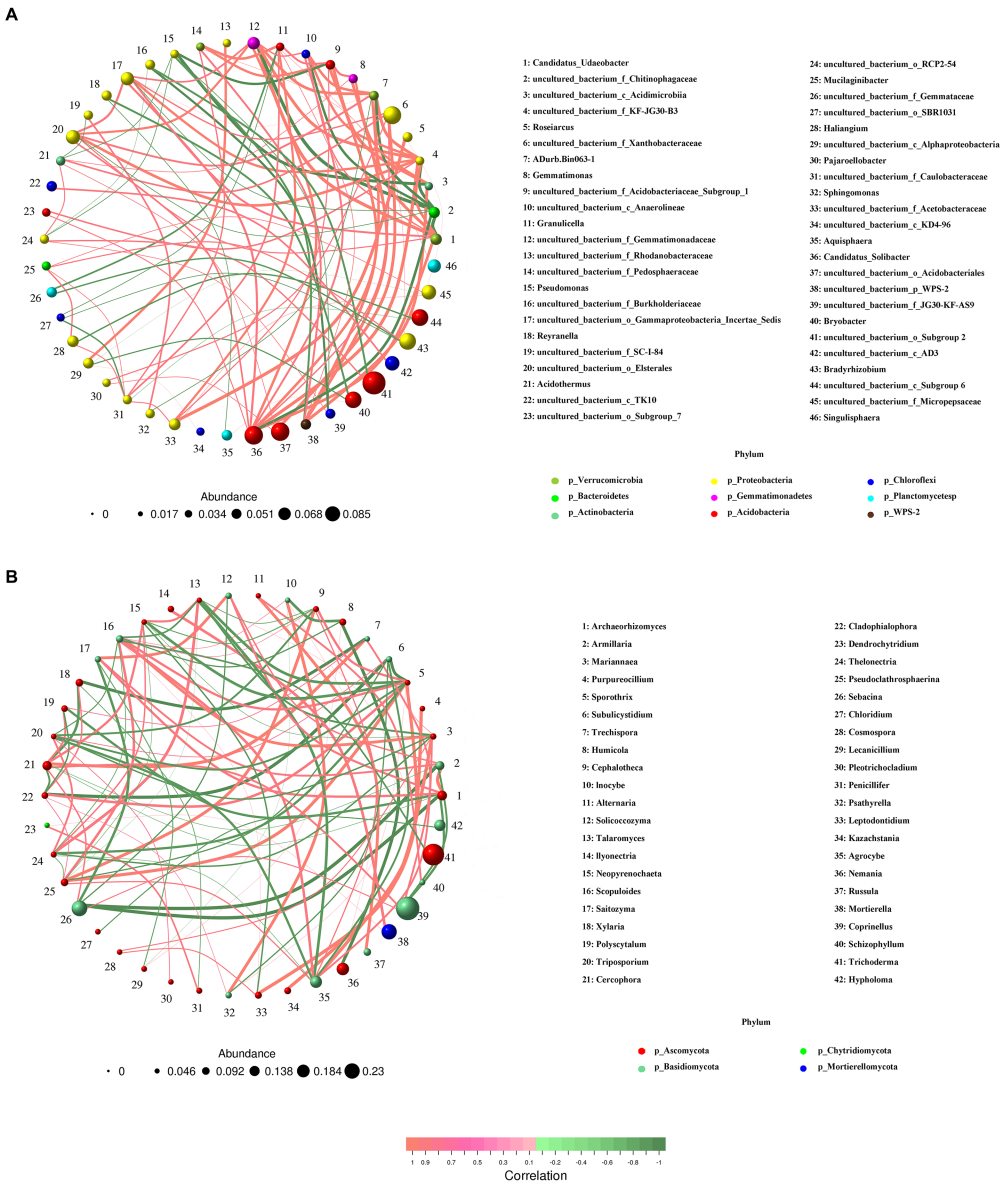
Co-occurrence analysis of soil bacterial **(A)** and fungal **(B)** communities in soils around *G. eleta* symbiosis with *Armillaria* strains.

Networks were constructed, respectively, for bacteria and fungi, and significant correlations were compared. We found that bacterial co-occurrence networks were larger, more connected, and more modular than those in fungal networks, and a larger proportion of bacterial OTUs was included in co-occurrence networks than that of fungal OTUs (Figure 8). In particular, the network for bacteria was composed of 46 nodes and 100 edges, and the modularity was 0.58; all nodes were assigned to 9 phyla. For the fungal community, the network presented 42 nodes and 100 edges; all these nodes were assigned to 4 phyla, i.e., Ascomycota, Basidiomycota, Chytridiomycota, and Mortierellomycota. Moreover, compared with Basidiomycota (14 nodes), Ascomycota (26 nodes) had a more complex correlation with different *A. gallica* strains.

## Discussion

The demand for *G. elata* production is expected to increase further in the future as a result of health preservation and wild resource shortages. Such concerns have led to the development of artificial cultivation of *G. elata,* in which *Armillaria* strains are functionally important factors in affecting the growth and yield of *G. elata*. Soil microbial communities are responsible for nutrient cycling and plant growth; these communities could be affected by abiotic or biotic factors, particularly interactions with other microorganisms (Singh et al., 2009; Kubiak et al., 2017). For these reasons, there has long been interest in selecting the optimum *Armillaria* strains to co-plant with *G. elata* in a variety of environments and disentangling the mechanisms behind the high yield of *G. elata*. This study is one of the few attempts to provide insight into the yield of *G. elata* co-planted with different *Armillaria* strains and the microbiome influenced beyond this interaction.

### The effect of *Armillaria gallica* strains on the yield of *Gastrodia elata*

The current study indicated that cultivation of *A. gallica* strains collected from other regions, especially Yunnan (YN strain), can significantly increase the yield of *G. elata* compared with that of the original Guizhou local strain (Figure 2A). Our finding is consistent with previous research in which four strains of *Armillaria* were identified as *A. gallica* groups, whereas co-planting of these *A. gallica* strains with *G. elata* resulted in significant differences in yield and active ingredient content of *G. elata* (Liu et al., 2019). However, how did that happen if the identification of *Armillaria* strains indicated that they were in close relation to *A. gallica* (Figure 1)? Beyond the classification results of *Armillaria* strains, a possible explanation is that the robust growth condition (Figure 1A) and greater tensile strength of mycelia in YN strain (Figure 2B) contributed to the high yield of *G. elata* compared with others, which is also evidenced by previous studies that the optimum *Armillaria* strains that can greatly increase the yield of *G. elata* are typically the ones with stronger branches of mycelia (Chen et al., 2004; Wang et al., 2020). Therefore, the tensile strength, which can be a measurement of the thickness and strength of *Armillaria* mycelia, was tested at harvest time, and the results also confirmed that the stronger the *Armillaria* branches, the higher the yield of *G. elata* that is harvested (Figure 2). In the meantime, given that *Armillaria* mycelia are generally considered a special morphological adaptation of this fungus to different environmental conditions (Garett, 1960; Morrison, 2004), the migration of YN, AH, and SX strains from their local habitat partly triggered their strong growth of mycelia. On the contrary, the adaptability of GZ strain to the local condition and its weak growth of mycelia might lead to its poor performance when co-planted with *G. elata* (Sun et al., 2009). Besides that, the great variation in soil bacterial and fungal communities arising from different *A. gallica* strains may also play vital roles in controlling the yield of *G. elata*.

### Response of soil bacterial and fungal communities to exotic *Armillaria gallica* strains and potential benefits on the growth of *Gastrodia elata*

Available studies have shown that the impacts of symbiotic mycorrhizal fungi on the diversity and composition of the soil rhizomicrobiome may induce changes in the ecological environment of soil microorganisms and, potentially, increase stress tolerance and yield of plants (Doubková et al., 2012; Cosme and Wurst, 2013; Selosse et al., 2022). In the current study, we observed that the GZ strain induced great changes in the bacterial community (Figures 3A,B, 4A,B, 5A), while the YN strain had a much stronger impact on the fungal community (Figures 3C,D, 4C,D, 5B). Moreover, a diverse variation and more complex fungal community composition were observed across all soil samples in responding to different *A. gallica* strains (Figures 6, 7). In a co-culture study, Collins et al. (2013) revealed the defensive and potentially offensive nature of *Armillaria* members and their ability to influence other microbes. Thus, it is acceptable that greater variation was observed in the fungal community induced by *A. gallica* strains, considering that soil fungal communities are more sensitive and respond more quickly to a variety of stimuli, including biological disturbance (Xiao et al., 2014).

As also reported, the *Armillaria* members are known as wood-rotting fungi (Brundrett, 2002), along with an ability to secrete wood-decomposition enzymes such as glycoside hydrolases. When *A. gallica* exists in soil and attempts symbiosis with *G. elata*, it will typically secrete enzymes to penetrate plant cell walls. The enzymes can mineralize or decompose most plant cell wall polymers into simple compounds (Floudas et al., 2012; Purahong et al., 2016), which increase their accessibility to other organisms. A previous study conducted with five *Armillaria* isolates reported a positive effect of an excellent *Armillaria* strain on the promotion of quality and yield of *G. elata* Bl. f. glauca in Changbai Mountain (Yu et al., 2022). The researchers found that excellent *Armillaria* strains increased the yield of *G. elata* Bl. f. glauca by improving the soil microbial environment, especially by increasing the relative abundances of some beneficial genera such as *Mortierella*, *Agrocybe*, and *Armillaria*. In accordance with this study, the high yield of *G. elata* co-planted with the YN strain was accompanied by higher relative abundances of *Mortierella*, *Nemania*, *Agrocybe*, *Archaeorhizomyces*, *Armillaria*, *Pseudoclathrosphaerina*, *Scopuloides*, *Leptodontidium*, *Cladophialophora*, *Psathyrella*, and *Saitozyma* compared with these in other *A. gallica* strains (Figure 7B). Species of the genera *Mortierella*, *Nemania*, and *Leptodontidium* were commonly reported as wood-rotting members and are sensitive to readily accessible carbohydrates (Tang et al., 2007; Lindahl et al., 2010; Werner et al., 2016). Their strong co-occurrence with the YN strain implied that they were candidate fungal taxa that might be involved in the high activity of *A. gallica*. Accordingly, *Archaeorhizomyces* species are non-pathogenic plant root and rhizosphere-associated fungi that commonly harbor deeper soil horizons with low pH and high nutrient turnover (Rosling et al., 2011; Carrino-Kyker et al., 2016). Previous findings suggest that correlations between *Archaeorhizomycetes* and other fungal taxa indicate that *Archaeorhizomycetes* might benefit from carbohydrates or nutrients (Pinto-Figueroa et al., 2019) and may also have potential associations with plant growth (Schadt et al., 2003). Similarly, species of the genus *Cladophialophora* possessed superior growth promotion activities as well as disease suppression, which significantly increased the growth parameters of strawberry plants (Harsonowati et al., 2020). As a result, the combination of yields of *G. elata* and responses of these fungal genera due to *A. gallica* strains in our study may reflect the potential performance of biological and ecological functions in soil fungal communities.

It is well known that the interactions of interspecific fungi are complex and dynamic processes (Awan and Asiegbu, 2021). Co-occurrence networks indicated that bacteria such as *Candidatus_Solicabcter*, *bacterium_f_Gemmatimonadaceae* were highly central and connected (Figure 8A), suggesting that they might drive the observed *A. gallica*-induced changes in bacterial networks. Additionally, for fungal networks, the genera *Sporothrix* (belonging to Ascomycota) and *Scopuloides* (belonging to Basidiomycota) all interacted with 12 genera, including 7 positive interaction genera and 5 negative interaction genera, implying their important roles in shaping fungal community under such complex environmental conditions. Based on previous studies, soil bacterial communities are less resistant but more resilient to disturbance than fungal communities (de Vries et al., 2018), and changes in functional taxa can alter the response of microbial communities to disturbance and their symbiotic relationships with host plants (Banerjee et al., 2018). In the current study, shifts in the relative abundance of dominant taxa (Figures 6, 7) might later facilitate the stability of networks (Figure 8), and consequently the assemblage of fungal communities in soil substrates and response of soil fungi to different *A. gallica* strains. Besides that, soil physicochemical properties or nutrient cycling were expected to be altered by symbiotic fungi (Finlay, 2004; Veresoglou et al., 2012), and correlations can be tested between environmental factors and microbial community structure. However, measurements of soil physicochemical properties were not conducted due to the slight amount of soil around *G. elata*, which is a limitation of this study. Further study is needed to discuss the changes in soil physicochemical properties arising from *A. gallica* strains and the correlations between them.

The current study emphasizes that soil fungi rather than bacteria were more easily modified by exotic *A. gallica* strains, and that might have a potentially beneficial impact on the growth and yield of *G. elata*. Specifically, the dominant advantage of the YN strain was evidenced by its role in triggering changes in the soil fungal community, especially relative abundances of some beneficial genera such as *Mortierella*, *Agrocybe*, *Neminia*, *Armillaria*, and so on when cultivated in Guizhou Province. Although our study identified these potentially beneficial genera accompanied by a high yield of *G. elata*, further support is needed to confirm the potential functions and ecosystem services of these soil microbes by isolating and proving their roles in suppressing or fostering *A. gallica* in a co-culture experiment or mutualistic trial study with *G. elata*.

## Conclusion

The current study revealed that under field conditions in *G. elata* and *A. gallica* symbiotic systems, the yield of *G. elata* significantly differed among *A. gallica* strains collected from several regions. In addition, the diversity, structure, and composition of soil microbial communities, especially fungal communities, strongly changed in response to different *A. gallica* strains, which might have a beneficial effect on the yield of *G. elata*. This comprehensive understanding of the responses that govern the selection and activity of microbial communities by *A. gallica* strains can be committed to practical management and provide us with new opportunities to increase the yields of *G. elata*.

## Data availability statement

The datasets presented in this study can be found in online repositories. The names of the repository/repositories and accession number(s) can be found at: https://www.ncbi.nlm.nih.gov/, PRJNA951876; https://www.ncbi.nlm.nih.gov/, PRJNA951884.

## Author contributions

YW: funding acquisition, methodology, formal analysis, writing—original draft preparation. JX: funding acquisition, methodology, and visualization. QY: funding acquisition, methodology, writing—review and editing. LG: conceptualization and supervision. CX: methodology and software. CY: formal analysis and data curation. LL: resources and data curation. WJ: conceptualization and supervision. TZ: conceptualization, funding acquisition, and supervision. All authors contributed to the article and approved the submitted version.

## Funding

This study was supported by the Guizhou Provincial Basic Research Program [Natural Science, project No. Z.K. (2021) General 523], the National Natural Science Foundation of China (projects No. 81960694 and No. 32060080), the China Agriculture Research System (CARS-21), the High-level Innovative Talents Program of the Guizhou Province of China [Qian Ke He Platform and Talent (2018) 5638–2], and the Science and Technology Project of the Guizhou Province of China [Qian Ke He Platform and Talent (2019) 5611].

## Conflict of interest

The authors declare that the research was conducted in the absence of any commercial or financial relationships that could be construed as a potential conflict of interest.

## Publisher’s note

All claims expressed in this article are solely those of the authors and do not necessarily represent those of their affiliated organizations, or those of the publisher, the editors and the reviewers. Any product that may be evaluated in this article, or claim that may be made by its manufacturer, is not guaranteed or endorsed by the publisher.

## Footnotes

^1^https://blast.ncbi.nlm.nih.gov/Blast.cgi

^2^https://www.who.int/medicines/publications/traditional/gacp2004/en/

^3^version 7.1, http://drive5.com/uparse.

^4^RDP, V.2.2, http://rdp.cme.msu.edu/.

^5^www.biocloud.net

## Supplementary material

SUPPLEMENTARY FIGURE S1 Collection sites of *Armillaria* rhizomorphs from four main producing regions of *G. elata*.

SUPPLEMENTARY FIGURE S2 The conditions of *Armillaria* strains packets used for *G. elata* artificial cultivation. **(A)**, **(B)**, **(C)**, and **(D)** represent *Armillaria* of YN strain, GZ strain, SX strain, and AH strain respectively.

SUPPLEMENTARY FIGURE S3 The conditions of *Armillaria* strains mycelia at the harvest time. **(A)**, **(B)**, **(C)**, and **(D)** represent *Armillaria* of YN strain, GZ strain, SX strain, and AH strain respectively.
