# Supplementary figures and images for: Effect of symbiotic fungi-Armillaria gallica on the yield of Gastrodia elata Bl. and insight into the response of soil microbial community
Source: Front Microbiol. 2023 Sep 7;14:1233555. doi: 10.3389/fmicb.2023.1233555 (PMC10512952; doi:10.3389/fmicb.2023.1233555)

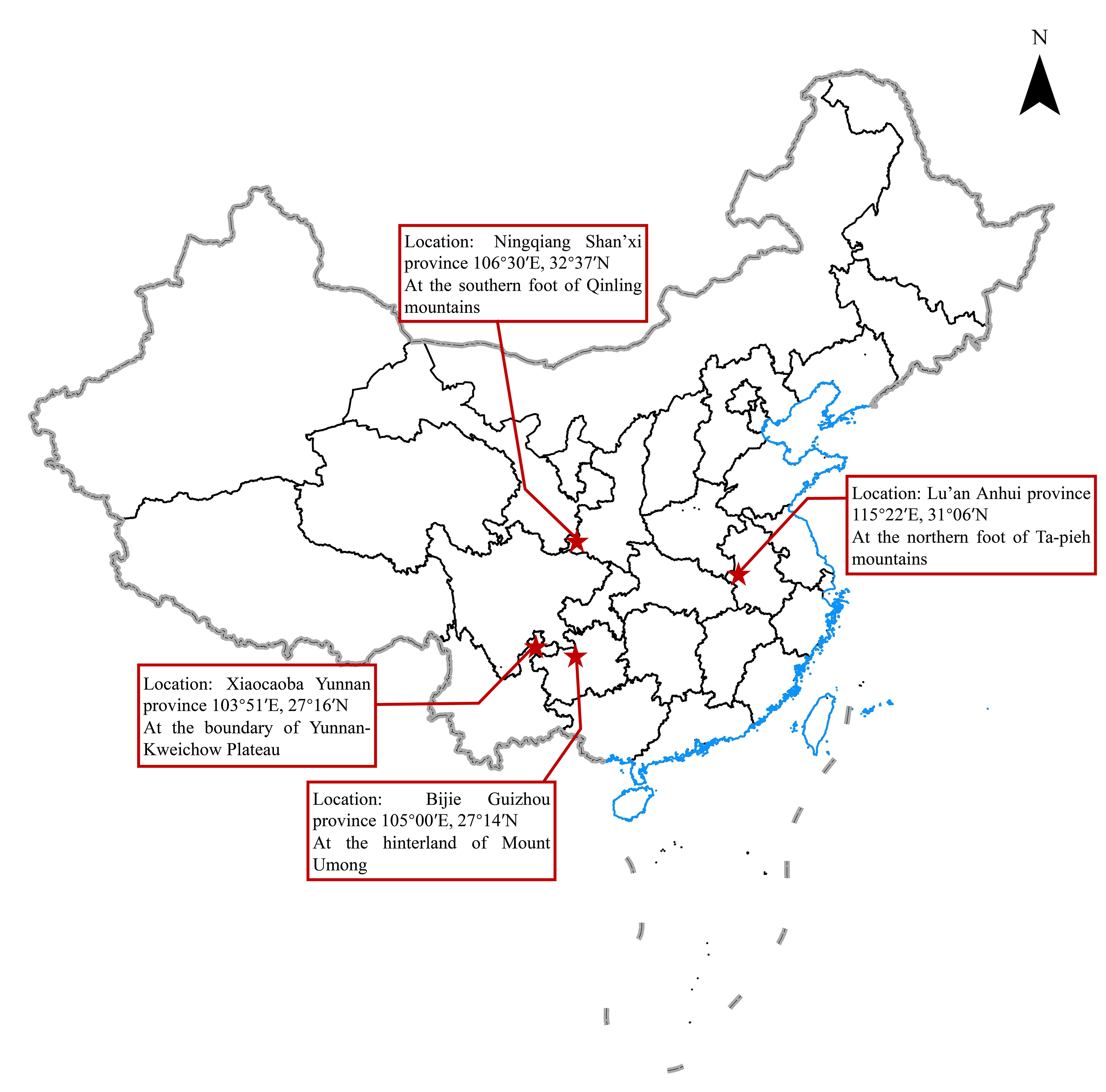

Supplement: SUPPLEMENTARY FIGURE S1 — Collection sites of Armillaria rhizomorphs from four main producing regions of G. elata. [file Image_1.TIF]

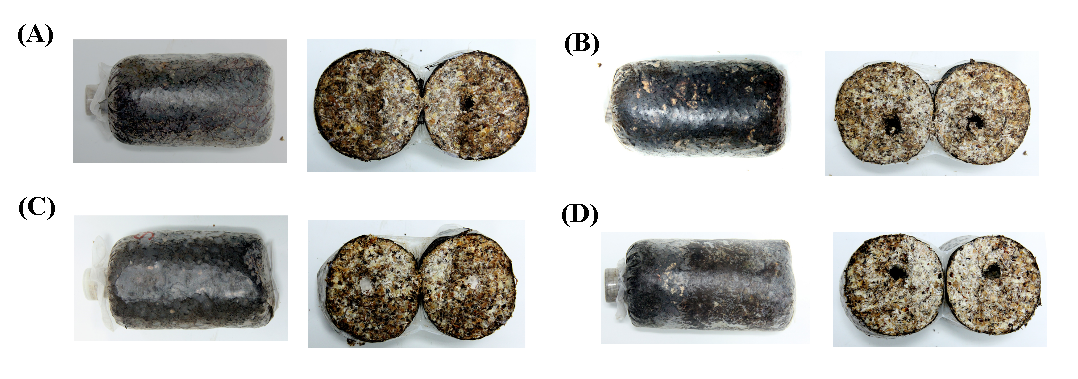

Supplement: SUPPLEMENTARY FIGURE S2 — The conditions of Armillaria strains packets used for G. elata artificial cultivation. (A), (B), (C), and (D) represent Armillaria of YN strain, GZ strain, SX strain, and AH strain respectively. [file Image_2.TIF]

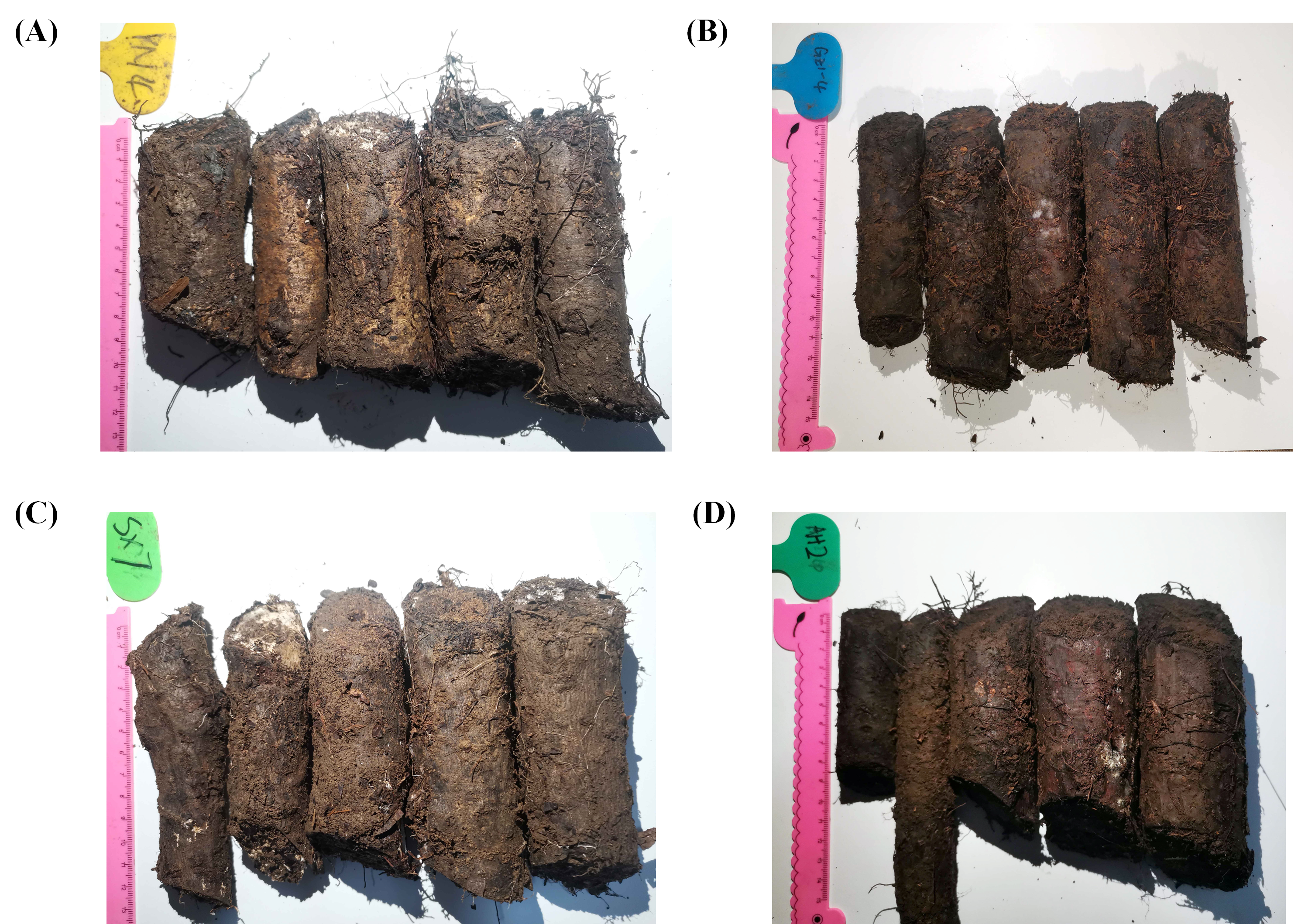

Supplement: SUPPLEMENTARY FIGURE S3 — The conditions of Armillaria strains mycelia at the harvest time. (A), (B), (C), and (D) represent Armillaria of YN strain, GZ strain, SX strain, and AH strain respectively. [file Image_3.TIF]
